# Translational Medicine in the Era of Social Media: A Survey of Scientific and Clinical Communities

**DOI:** 10.3389/fmed.2019.00152

**Published:** 2019-07-03

**Authors:** Elena Sandalova, Julie G. Ledford, Mani Baskaran, Suzan Dijkstra

**Affiliations:** ^1^Danone Nutricia Research, Singapore, Singapore; ^2^Utrecht Institute for Pharmaceutical Sciences, Utrecht University, Utrecht, Netherlands; ^3^Department of Cellular and Molecular Medicine, University of Arizona, Tucson, AZ, United States; ^4^Singapore National Eye Centre, Singapore Eye Research Institute, Singapore, Singapore; ^5^The Ophthalmology & Visual Sciences Academic Clinical Program (EYE-ACP), Duke-NUS Graduate School, Singapore, Singapore; ^6^Medical Student, Utrecht University, Utrecht, Netherlands

**Keywords:** social media, translational medicine, *Chatbot*, facebook, twitter

## Abstract

**Background:** The integration of new scientific discoveries into clinical practice costs considerable time and resources. With the increased use of social media for scientific communication, new opportunities arise to “bridge the gap” in translational medicine. The present study aimed to investigate how medical professionals access scientific information and understand their view on the role of social media in translational medicine.

**Methods:** A questionnaire regarding (i) the use of social media for scientific updates, (ii) the opportunities and challenges of social media for translational medicine, (iii) social media function *Chatbot*, and (iv) participant demographics was developed. The survey link was posted online from February, 2018, until April, 2018.

**Results:** A total of 555 professionals responded to the survey. Respondents identified themselves predominantly as researcher/scientists (27%) or medical/biomedical students (15%). The majority of participants was employed at a university or research institute (59%), and most practiced either in Europe (48%) or in Asia (37%). Seventy-eight percent of respondents reported receiving most of scientific news and updates via non-social media options, such as journal websites and newspapers. Fifty-one percent of respondents believed that social media could contribute to closing the gap between scientific discovery and translation to medical application. The most crucial opportunity created by social media was found to be “connecting the right scientist to the right clinician.” Participants rated “the translation of scientific finding to clinical practice is too fast before the safety is properly demonstrated” as the most crucial challenge. Half of the respondents were aware of their institutions policy on the professional use of social media. Only 2% of respondents had previously used *Chatbot*.

**Conclusions:** Overall, medical professionals were positive about the idea that social media could contribute to the progress of translational medicine. However, it is clear that they are still being cautious about using social media for professional purposes. To fully harness the potential of social media on translational medicine, the medical community needs to be provided with educational programs, guidelines, and support infrastructure within social media.

## Introduction

The integration of new scientific discoveries, whether they be into clinical practice or into the pharmaceutical or nutritional industries, costs considerable time and resources. Most biomedical research institutions still excel in basic research. However, less effort is given to the dissemination of information to the general public. It is evident that a gap exists between the biomedical research community and patients in need of their discoveries (1). Although multiple organizations have dedicated efforts to reduce the time to implement new knowledge and research findings (2), the translation of progress made by basic, preclinical researchers into new therapies that benefit patients remains a long, difficult, and expensive process (3, 4).

Over the past decades, social media (SoMe) have aimed to connect people from all over the world, with popular SoMe forum Facebook's mission statement even being “to bring the world closer together” (5). The presence of medical journals on social media, sharing of their articles, and appearance of multiple entities that aim to explain the scientific findings to public could give rise to a new opportunity to “bridge the gap” in translational medicine. In addition to connecting medical professionals, patients and other individuals can share scientific information on SoMe as well, often adding their view on it. Moreover, large SoMe forums such as Facebook, Twitter, Instagram, and LinkedIn allow patients to organize themselves and raise both awareness and funds for topics for which they share a common interest (6, 7). SoMe also serve as a source of information for patients; thus, it is crucial for the medical community to be aware and influence the quality and assess the validity of the posted information (8). The measures to evaluate the effect of the social media in engagement of a population of interest are still being discussed and developed. Generally, the number of likes is used as the most frequent type of assessment of engagement (9); however, there are some concerns with such approach. There is a possibility that people like posts for various reasons and are not accessing or reading the content. Thus, various new measures need to be developed in order to improve the assessments of engagement by social media.

Several studies have assessed the use of social media by professionals. Mostly professionals use SoMe for personal rather than professional purposes (10, 11). There are efforts to call scientists into action to have a greater presence on SoMe as professionals (12). However, there are also skeptics that warn against potential pitfalls of social media (13, 14). Even though SoMe platforms have been around for decades, the medical society is late in embracing the use of them in a professional setting. However, SoMe are here to stay! Thus, education about the appropriate use of social media; implementation of policies from government, institutions, and professional societies; and full utilization of SoMe functions is crucial for bridging the gap between scientific discovery and clinical applications and involving the patients in all stages of translational research. Introduction of these new platforms and applications that could help to screen the information and interpret it could benefit translation of research and support clinicians and patients to find what they need in the enormous sea of facts and news.

Automated conversational tools, or *Chatbots*, have begun to receive interest in the healthcare and research spaces. *Chatbots* often accompany SoMe, however, can function as independent tools on any digital platform. A recent quick search on PubMed for the term “Chatbots” revealed 31 publications, starting from as early as 2011. While clearly a very new topic, the use of *Chatbots* is picking up in both medical practice and research studies. Such efforts are being made to create and use *Chatbots* in research and in practice, particularly in the field of psychiatry, such as mental health (15, 16); medication management (17); and behavioral interventions in obesity (18). Pereira et al. (19) conducted a search on the *Chatbots* in healthcare aiming at behavioral change. The study revealed 30 articles mainly focusing on nutritional and neurological disorders. Overall, there are multiple efforts to create *Chatbots* to support patients and healthcare professionals. However, there are no broadly used tools for this purpose. Thus, in addition to the questions regarding SoMe, this study aimed to create a better understanding about the awareness of the medical and research professionals about *Chatbots* and whether they have experience with this technology in their practice.

In order for translational medicine professionals to utilize social media to their best potential, it is necessary to better understand how participants in this field, which include both researchers and medical professionals, perceive and use SoMe and their tools. Therefore, the aim of the present study was to investigate how scientists and clinicians access scientific information and provide insights into their view on the role of social media in translational medicine.

## Methods

### Survey Development

The survey was designed with the following research questions in mind: “Do professionals use SoMe for scientific updates?,” “Do professionals think that SoMe can contribute to the progress of translational medicine?,” “How do professionals rate potential opportunities and challenges that SoMe bring to translational medicine?,” and “Do professionals use social media function *Chatbot?*” In the development of the survey, the authors aimed to include no more than 15 questions, so that it could be completed in <5 min and increase the likelihood of participation in the survey. Questions on profession, workplace, age group and geographic location were included in order to understand if professionals' attitude to SoMe is affected by any of these factors. A total of 11 questions regarding participant demographics and the research questions were developed based on consensus by all authors. An overview of the survey questions is provided in Table 1. The full survey including introductory text is presented in the Supplementary Document 1. In adherence to the guidelines of the SingHealth Centralized Institutional Review Board (CIRB), the nature of this study met with the criteria to be exempt from CIRB review.

**Table 1:** Questions and answer options of the survey.

	**Questions**	**Dropdown selection/Answer options**
1	What best describes your position/profession?	a. Research assistant b. Researcher/scientist c. Professor d. Medical professional e. Clinician-scientist f. Medical/Biomedical student g. PhD student h. Management position (in industry, institution) i. Other (Please specify)
2	Where do you work/study?	a. University or research institute b. Academic hospital c. Non-academic hospital d. Industry (pharma, nutrition, medical device, etc.) e. Other (Please specify)
3	What is your age range?	a. Below 20 b. 20–30 c. 31–40 d. 41–50 e. Above 50
4	Where do you work?	a. Europe b. North America c. South America d. Africa e. Asia f. Australia
5	What sources do you use most to follow scientific news? (multiple answers possible)	a. Journal's websites b. Newspapers and/or news applications on mobile devices c. Update emails from journals d. Updates from your institution (website, newsletters, etc.) e. Facebook f. Linkedin g. Twitter h. Other (Please specify)
6	Do you think that social media can contribute to closing the gap between scientific discovery and its translation to medical application?	a. Yes b. No c. Maybe
7	If yes or maybe, which are the most crucial opportunities social media create? (rate 1–6)	a. Faster dissemination of scientific information b. Broader dissemination of scientific information c. Allowing open criticism of scientific discoveries d. Connecting the right scientist to the right clinician e. Facilitating the recruitment in clinical studies f. Facilitating surveys/online studies g. Other (Please specify)
8	If yes or maybe, which are the most crucial challenges social media create? (rate 1–4)	a. Distribution of fake news and incorrect conclusions b. Distribution of fraud c. Public over-reaction of un-confirmed findings d. The translation of scientific finding to clinical practice is too fast before the safety is properly demonstrated e. Other (Please specify)
9	Are you familiar with chatbot (Robot human-like conversational tool used on social media messaging platform)?	a. Never heard about it b. Yes, I've heard about it but I've never used it c. Yes, I use/have used this tool
10	Do you use chatbot (Robot human-like conversational tool used on social media messaging platform) for your work?	a. Yes b. No
11	Are you aware about your institutions policy on the professional use of social media?	a. Yes b. No

### Survey Distribution

The survey was uploaded to online survey platform *SurveyMonkey* using the ADVANTAGE Team plan and was distributed via a number of forms of communication, represented in Table 2 and Supplementary Document 2. In addition, the link could have been shared by respondents via their personal social networks and emails, which we would have been unable to track. Thus, the total number of approached professionals was estimated at 57,468.

**Table 2:** Survey distribution.

**Source^a^**	**Estimated number of individuals reached**
*Singapore Women in Science*	310
*Eureka Institute* alumni	241
*Apollo Society* chapters in Utrecht	30
*Apollo Society* chapters in Toronto	100
*SingHealth*	22,698
Institute of Medical Biology A^*^Star	300
*Karolinska Institute* facebook page	30,566
*Utrecht University* Medical students facebook pages and website	1,800
*Utrecht Institute for Pharmaceutical Sciences*	58
Personal *LinkedIn accounts, views*	763
Personal *Twitter accounts, views*	27
Personal *Facebook accounts, views*	81
Emails to personal professional connections	494
Total	57,468

a *The survey was distributed to personal contacts, social media forums, and through several scientific organizations. A brief description and the websites of these organizations are provided in Supplementary Document 2*.

The survey was launched on the 5th of February, 2018. The survey results were downloaded on the 25th of April, 2018.

### Data Analyses

Prior to survey conduct the margin error was set to be below 5% with 95% confidence interval. We estimated that the medical and biomedical scientific community consists of 10^7^ doctors and 10^7^ biomedical scientists (20). As the survey outcomes are based on proportions and assuming the most conservative standard deviation when the proportion is 50%, a minimum of 385 respondents would be required based on an online calculator with a 5% margin of error (21, 22).

The analysis was performed using *SurveyMonkey* filtering and comparing tools and *Graphpad prism* (version 6). The number of respondents was converted into proportions and these were then compared. Subgroup analyses were performed for profession, workplace, age group and geographical location. The total number of individuals in the group was set as 100 percent and the responses for the respective question were compared. Bonferroni correction for multiple group comparison was applied.

## Results

In the 11 weeks that the survey link was online, the total number of respondents reached 555. As explained in the methods section, a minimum of 385 respondents was calculated to lead to a margin error <5%. The response rate in this study of 555 respondents led to a margin error of 4.16% (22).

### Demographics of Survey Respondents

An overview of the demographic characteristics of the survey respondents is provided in Table 3. Of those individuals that participated in our study, the highest percentage identified themselves as researcher/scientist (27%), followed by medical/biomedical student (15%) and medical professional (13%). The majority of participants were employed at a university or research institute (59%). The age of survey participants was grouped into several categories. The highest proportion of participants were between the ages of 31–40 years (35%), followed by 20–30 year olds (29%). While surveys were distributed through contacts world-wide, most participants indicated that they worked in either Europe (48%) or Asia (37%). There were no respondents from Africa.

**Table 3:** Respondents characteristics.

**Characteristics**	***N* = 555**
**Profession**, ***n*** **(%)**	
Researcher/scientist	150 (27)
Medical/biomedical student	82 (15)
Medical professional	72 (13)
Professor	56 (10)
Management position	53 (10)
Clinician-scientist	43 (8)
Ph.D. student	38 (7)
Research assistant	27 (5)
Other	32 (6)
Participant skipped question	2 (0)
**Workplace**, ***n*** **(%)**	
University or research institute	329 (59)
Academic hospital	111 (20)
Industry	68 (12)
Non-academic hospital	26 (5)
Other	21 (4)
**Age group**, ***n*** **(%)**	
Below 20	8 (1)
20–30	161 (29)
31–40	192 (35)
41–50	130 (23)
Above 50	64 (12)
**Geographical location**, ***n*** **(%)**	
Europe	268 (48)
Asia	203 (37)
North America	64 (12)
Australia	11 (2)
South America	7 (1)
Africa	0 (0)
Participant skipped question	2 (0)

### Sources of Scientific News

**Figure 1:**
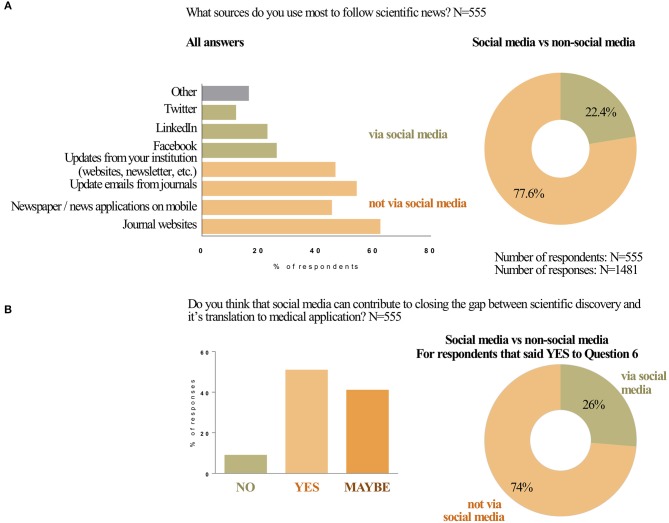
Social media in translational medicine. Use of social media for scientific updates **(A)**. What sources do you use most to follow scientific news? Responses to Question 5 of the survey are plotted in the bar graph. For the doughnut graph the responses were combined for all social media (Twitter, Facebook, and Linkedin) and for non-social media sources (Journal's websites, Newspapers and/or news applications on mobile devices, Update emails from journals, Updates from your institution). Can social media contribute to closing the gap between scientific discovery and its translation to medical application **(B)**? Doughnut graph represents the use of social media (Question 5) by those who responded YES to Question 6.

One of the possible functions of SoMe is information sharing. In survey question 5, participants shared their use of SoMe vs. other resources to update themselves on scientific news. A majority of participants (77.6%) reported receiving most of scientific news and updates via non-social media options (Figure 1A). The most utilized non-social media outlets included journal websites, newspaper or news applications on mobile devices, update emails from journals and updates or newsletters from the professional's individual institution. Of the 22.4% of respondents that used social media as a means to receive scientific updates, participants relied on Facebook, Linkedin, and Twitter.

### Social Media in Closing the Gap Between Scientific Discovery and Its Translation to Medical Application

When asked if social media could contribute to closing the gap between scientific discovery and translation to medical application, half of the respondents (50.5%) said “yes,” while 41% answered “maybe” and 8.5% answered “no” (Figure 1B). When comparing these answers for subgroups, several differences were found to be statistically significant (i.e., had a *P*-value <0.05). Based on profession, significantly more researchers/scientists said “yes” then did professors, clinician-scientists and students. Significantly more researchers/scientists indicated that they believe SoMe can contribute to translational medicine compared to those who said “no,” while significantly more professors said “maybe” compared to those who said “yes” (Supplementary Figure 1A). Lastly, significantly more students said “no” compared to those who said “yes.” Answers differed for age groups as well. The most optimistic age group was the 31–40 year olds, where significantly more respondents said “yes” compared to those who said “no” (Supplementary Figure 1B). No significant differences in responses to this question were found between respondents working in different geographic locations or types of workplaces (Supplementary Figure 1C and data not shown).

### Opportunities and Challenges of Social Media in Translational Medicine

**Figure 2:**
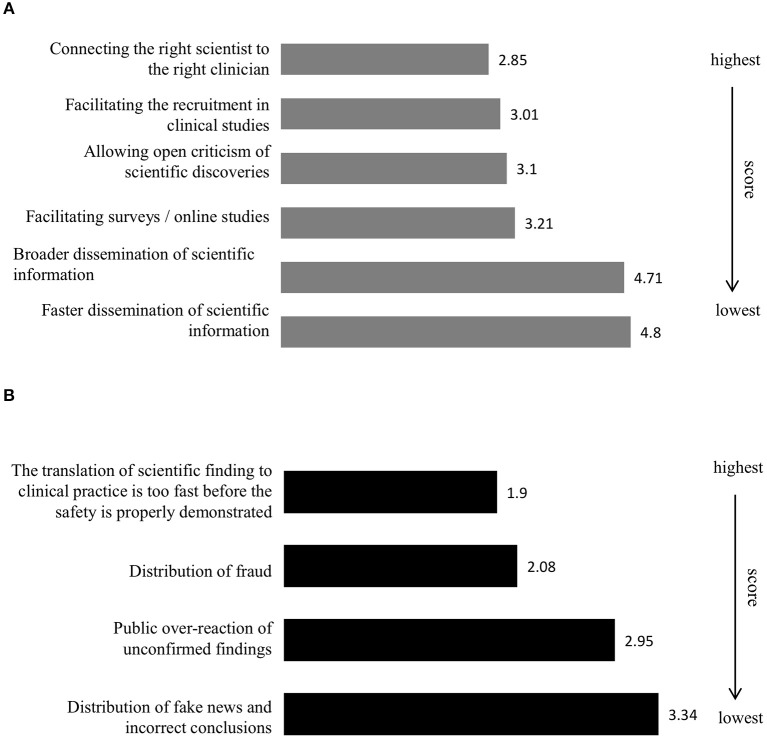
Opportunities and challenges of social media in translational medicine. The most crucial opportunities social media create **(A)** (Q7) [If you answered yes or maybe to the previous question, which are the most crucial opportunities social media create (rate with 1 being the highest)]. The most crucial challenges social media create **(B)** (Q8) [If you answered yes or maybe to question 6, which are the most crucial challenges social media create (rate with 1 being the highest)]. Average score from all the respondents for each statement have been calculated and presented in the bar graph.

The respondents were asked to rate the most crucial opportunities that SoMe create, with a rating of 1 indicating the highest priority and 5 indicating the lowest priority. They scored “connecting the right scientist to the right clinician” as the most crucial with an average score of 2.85 (Figure 2A). “Facilitating the recruitment in clinical studies,” “allowing open criticism of scientific discoveries,” and “facilitating surveys/online studies” scored 3.01, 3.1, and 3.2, respectively. The potential opportunities found to be least crucial were “broader dissemination of scientific information” and “faster dissemination of scientific information,” scoring 4.71 and 4.8, respectively.

The respondents scored “the translation of scientific finding to clinical practice is too fast before the safety is properly demonstrated” and the “distribution of fraud” as the most crucial challenges with average scores of 1.9 and 2.08, respectively (Figure 2B). “Public over-reaction of unconfirmed findings” and “distribution of fake news and incorrect conclusions” were believed to be less crucial at 2.95 and 3.34, respectively.

### Institutional Policy on the Professional Use of Social Media

**Figure 3:**
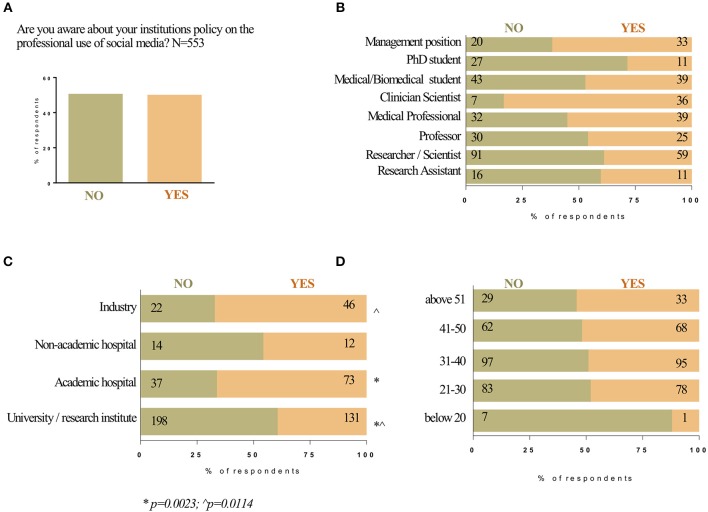
Awareness about own institution's policy on use of social media. The responses to Question 11, “Are you aware about your institutions policy on the professional use of social media?” are shown on the column graph in percentages **(A)**. Institutions policy and occupation **(B)**. Institutions policy and workplace **(C)**. Institutions policy and age **(D)**. The bar graph illustrates the percent of each occupation/workplace/age group of the respondents that answered with NO (olive bar) and YES (light orange bar) to Question 11. The numbers at each side of the bar indicate the number of responses for NO/YES for each occupation/workplace/age group.

Of the 555 participants, responses were split ~50 and 50% with those that were aware and those that were not aware of their specific institutions policy on the professional use of social media (Figure 3A). Those that were most aware were clinician-scientists and respondents in management positions (Figure 3B). Those that were the least aware were PhD students and researcher/scientists. Overall, those employed by industry or academic hospitals were more likely to be aware of the institutions policy on SoMe usage compared to those in non-academic hospitals and university/research institutes (Figure 3C). There was a similar level of understanding (~50:50) among all age groups except in the under 20 group, which was the smallest age group; in the under 20 group only 1 out of 8 respondents was aware of the social media policy of their institution (Figure 3D).

### Familiarity With and Usability of Chatbot

**Figure 4:**
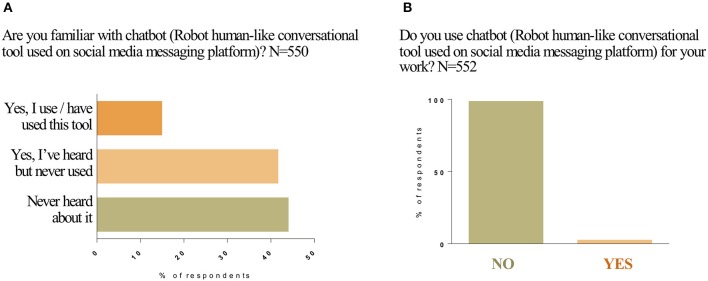
Awareness and use of *Chatbot*. Are you familiar with Chatbot **(A)**? Do you use Chatbot for your work **(B)**? Responses to Question 10 were plotted on the column graph in percentages.

Participants were asked if they were familiar with *Chatbot*, a robot-like conversational tool used on social media messaging platforms. *Chatbot* is an example of functions within SoMe that can be used by professionals to search for information. Of the 550 participants that answered this question, 45% responded that they had “never heard about it (i.e., *Chatbot*);” while 43% answered “yes, I've heard of it but never used,” and only 17% responded “yes, I use/have used this tool.” Five hundred and fifty-two of the participants responded to the question “do you use *Chatbot* for your work?” A majority of 98% of respondents answered “no” to this question (Figure 4 and Supplementary Figure 2).

## Discussion

The last decade has completely re-shaped the way in which we communicate. It has become clear that internet-based communication is growing and all fields of life have to adapt to its use, including the medical and research communities. Communication on SoMe forms a large part of internet-based communication and plays a crucial role in science information sharing, discussion and implementation of scientific discoveries. However, we are still learning how to properly use SoMe, while also assessing the associated risks and benefits. In order to utilize social media outlets to their best potential, while minimizing their disadvantages, it is important to first understand how social media are being perceived and utilized by members of the translational medicine community.

The survey respondents in this study—predominantly research scientists, medical professionals, and students—are still relying on conventional non-social media methods, albeit more often online, for reliable scientific news. Respondents speculated that the gap between discovery and translation could be bridged by SoMe, but at the same time feared that premature dissemination of results might be unsafe. Moreover, dissemination of fraud “fake” news was felt to be a problem. Tools such as *Chatbot*, which may help professionals fish for information on SoMe, seem to be utilized only minimally amongst the survey respondents. This could be for several reasons: there are still too few *Chatbot* tools that exist for research and/or medical advice or professionals are less aware about opportunities that *Chatbot* presents (23, 24).

In this study population, less than one-third of the medical community utilized SoMe for scientific news. This may suggest two things: (1) the medical community has not changed its way of looking for reliable scientific information or (2) the scientific journals have just started utilizing the power of SoMe in transmitting scientific information to professionals. In this study, specialized scientific social networking sites such as *ResearchGate* and *Mendeley* were not included in the popular list. However, among the answer “others,” only a few participants listed *ResearchGate, Medscape*, and *Google Scholar*.

Despite the smaller proportion of the scientific community relying on scientific news in SoMe more than half indicated that they “believed” that it has the potential to close the gap between scientific discovery and its translation to medical application. This may suggest that the society is in a transition phase between starting to explore the functions of SoMe and fully utilizing them professionally. Interestingly, the number of students in the survey that were optimistic in their belief that SoMe could close the gap between scientific discovery and translation to medical application was relatively small compared to researchers. We could not explain this phenomenon due to the small sample size and lack of additional data. If this finding is indeed true, it may be necessary to familiarize the student community with social media tools in translational aspects of medicine and consider adapting our education programs to include the use of SoMe training.

The favorite opportunity that the majority researcher/scientist respondents sought for social media to address was “to connect clinician and scientist,” which is an important step in translational research. It may also be a challenge with only 8% clinician scientists responding in this survey. This can be noted by policy makers in bridging the communities through better usage of SoMe platforms within institutes, across scientific communities, and the public. Embracing SoMe in disseminating knowledge and research in public health seems to be adopted by many scientific (9, 25, 26) and patient forums already (27). Dissemination of internal policies to the students and researchers seems a priority in this respect, as they were least aware of the SoMe usage rules within their institutes.

There are tools available for professionals to utilize for “recruitment and clinical trials,” such as *Chatbots* (12) and text mining approaches (28). However, the actual usage seems to be poor. While adaptation to such tools may be considered, caution should be exercised as these can also be subjected to trolling, privacy and other ethical issues (29). Any workshop or awareness program in this respect should engage ethical and technical experts to caution the “tech-naïve” medical professionals. It is no surprise that the “broader and faster dissemination of scientific knowledge” component of SoMe seems to be less appealing to a medical community. However, with more time spent on SoMe by the current and future generation, it may only be prudent for the scientific community to tap on this opportunity to disseminate new scientific information through SoMe in a reliable and realistic manner.

“Distribution of early clinical trials to patient community and false information” is undoubtedly the biggest challenge aspect surfacing in this survey, and it will prove to be a challenge that need to be tackled by the medical community in future (2, 13, 30). In this context, an active participation of the journals in disseminating such information, especially after subjecting the content to peer review before publication, may alleviate such issues.

The growth of platforms for interactions of professionals, such as *Labspaces, Sermo, DailyRounds, Among Doctors*, and others might be beneficial for the purpose of interaction of within professional groups (31). However, they do not integrate other professions or members of the public. Another approach would be to create pages or groups within bigger social media portals, mainly Twitter and Facebook (12, 32).

In the present survey study, the focus of the analysis was on the whole community of medical professionals and researchers. Some of the occupations might be under-represented, such as PhD students, clinician-scientists and research assistants (Table 3). Future studies should focus on these groups specifically to understand the use of SoMe. The present study was also limited by its low representation of professionals working in Africa and South America. It would be interesting to conduct a survey focusing on Africa in particular, as this is the continent with lowest internet and SoMe penetration, whereas North and South America have comparable internet and SoMe use (33, 34). The growing use of social media in Africa is an opportunity for dissemination of truthful information and engagement of the African community. Groups from trusted universities have the capacity of engaging new readers. Online educational programs for the use of social media would also be able to reach a bigger audience. Moreover, in this survey study, we focused on scientists and medical professionals. Clearly, there are other professions that contribute to translational medicine such as clinical study specialists, statisticians and data managers, patent attorneys, legal professionals who work with research and development, hospital and institutional administration, science communicators, patients, venture capitalists, and others. In order to complete the picture, future studies would need to assess the holistic relationship of all the people involved in the path of science creation and translation to medical applications, which will also include the end users' (i.e., patients) inputs.

The current study also puts social media to the test in conducting the actual research presented in this study. Only a small fraction of people whom the survey could potentially reach chose to participate (555 out of 57,468). Thus, future strategies for dissemination of such research which utilizes SoMe as the only outlet needs to consider the limitations for this method for dissemination. While common methods to bolster engagement include paid advertisement of the surveys, attracting influencers with significant followers and other innovative solutions, one must take into account that certain countries and age groups may not respond to such surveys on SoMe for a wide variety of unknown and unpredictable reasons.

Our study highlights that there is a clear need for specific educational programs and guidelines to be provided to the medical community in order for participants to harness the potential of SoMe on advancing discoveries and treatments in translational medicine. Such programs could include courses in universities dedicated to SoMe opportunities, pitfalls, and use. There should be courses with continual medical education (CME) credit points for educating the current workforce. In addition, SoMe could also be utilized for education purposes via scientific journals or university groups. Finally, encouraging more research in this area would also improve our understanding and help to grow the capable community to utilize SoMe to the full potential.

In conclusion, we found that the overall awareness of social media's role in translational medicine was realized by the medical community in this survey, but there seems to be lack of practical applications and utility. Educational programs and guidelines may provide the medical community with the tools to harness its potential.

## Ethics Statement

In adherence to the guidelines of the SingHealth Centralized Institutional Review Board (CIRB), the nature of this study met with the criteria to be exempt from CIRB review.

## Author Contributions

ES created the online survey and performed the statistical analyses. SD edited the manuscript. ES, JL, MB, and SD contributed to the survey development, data acquisition, and writing of this study.

### Conflict of Interest Statement

ES is employed by Danone Nutricia Research. The remaining authors declare that the research was conducted in the absence of any commercial or financial relationships that could be construed as a potential conflict of interest.

## Abbreviations

SoMe: social media.
