# Supplementary material for: Translational Medicine in the Era of Social Media: A Survey of Scientific and Clinical Communities
Source: Front Med (Lausanne). 2019 Jul 3;6:152. doi: 10.3389/fmed.2019.00152 (PMC6616126; doi:10.3389/fmed.2019.00152)

Supplementary Material

**Translational Medicine in the Era of Social Media: a Survey of Scientific and Clinical Communities**

**Elena Sandalova^1,2^, Julie G. Ledford^3^, Mani Baskaran^4,5^, Suzan Dijkstra^6^***

*** Correspondence:** Suzan Dijkstra: [Suzan@apollosociety.eu](mailto:Suzan@apollosociety.eu)

# Supplementary Figures and Tables

## Supplementary Document 1. Full survey including introductory text

### Introductory text

If you are a medical professional, an academic or industry scientist involved in biomedical research, a medical or biomedical student please takes a few minutes to fill in this survey. 

New scientific discoveries are published every day. There is a vast amount of new information and data related to medicine available every day. How does the scientific and medical community navigate through this sea of information? 

New knowledge generated by biomedical research is still taking a very long time to get integrated into clinical practice, be that a new potential drug, a new diagnostic tool or implementation of new protocols. Could social media help to close this gap and decrease the lagging time between new discoveries and improved treatment for patients?

Please help us by filling in this survey to gain better understanding of how scientific news is accessed by medical and research professionals and to find out what is the view of the medical and scientific community on the role of social media in translational medicine.

These data will be analysed by the following scientists:
Elena Sandalova, PhD, senior scientist, Danone Nutricia Research
Baskaran Mani, Asst Prof, DO DNB PhD, Clinician Scientist 
Remi Stevelink, MSc, MD and PhD student at University Medical Center Utrecht
Suzan Dijkstra, BSc, MD student at University Medical Center Utrecht, the Netherlands

Julie Ledford, PhD, Assistant Professor of Medicine, University of Arizona

### Full survey

|  | Questions | Dropdown selection / Answer options |
| --- | --- | --- |
| 1 | What best describes your position / profession? | a. Research assistant  b. Researcher / scientist  c. Professor  d. Medical professional  e. Clinician-scientist  f. Medical/Biomedical student  g. PhD student  h. Management position (in industry, institution)  i. Other (Please specify) |
| 2 | Where do you work / study? | a. University or research institute  b. Academic hospital  c. Non-academic hospital  d. Industry (pharma, nutrition, medical device, etc)  e. Other (Please specify) |
| 3 | What is your age range? | a. Below 20  b. 20-30  c. 31-40  d. 41-50  e. Above 50 |
| 4 | Where do you work? | a. Europe  b. North America  c. South America  d. Africa  e. Asia  f. Australia |
| 5 | What sources do you use most to follow scientific news? (multiple choices possible) | a. Journal’s websites  b. Newspapers and /or news applications on mobile devices  c. Update emails from journals  d. Updates from your institution (website, newsletters, etc)  e. Facebook  f. Linkedin  g. Twitter  h. Other (Please specify) |
| 6 | Do you think that social media can contribute to closing the gap between scientific discovery and its translation to medical application? | a. Yes  b. No  c. Maybe |
| 7 | If you answered yes or maybe to the previous question, which are the most crucial opportunities social media create? (rate with 1 being the highest) | a. Faster dissemination of scientific information  b. Broader dissemination of scientific information  c. Allowing open criticism of scientific discoveries  d. Connecting the right scientist to the right clinician  e. Facilitating the recruitment in clinical studies  f. Facilitating surveys / online studies  g. Other (Please specify) |
| 8 | Did you miss some opportunities stated in question 7? | a. No  b. Other (Please specify) |
| 9 | If you answered yes or maybe to question 6, which are the most crucial challenges social media create? (rate with 1 being the highest) | a. Distribution of fake news and incorrect conclusions  b. Distribution of fraud  c. Public over-reaction of un-confirmed findings  d. The translation of scientific finding to clinical practice is too fast before the safety is properly demonstrated  e. Other (Please specify) |
| 10 | Did you miss some challenges stated in question 9? | a. No  b. Other (Please specify) |
| 11 | Are you familiar with chatbot (Robot human-like conversational tool used on social media messaging platform)? | a. Never heard about it  b. Yes, I’ve heard about it but I’ve never used it  c. Yes, I use / have used this tool |
| 12 | Do you use chatbot (Robot human-like conversational tool used on social media messaging platform) for your work? | a. Yes  b. No |
| 13 | Are you aware about your institutions policy on the professional use of social media? | a. Yes  b. No |

## Supplementary Document 2. Sources used for distribution of the survey

| **Source** | **Website^a^** | **Description^b^** |
| --- | --- | --- |
| *Singapore Women in Science* | <https://www.singaporewomeninscience.org/> | “…an organisation of women who work and study in academia, patent law, business development, technology transfer, pharmaceutical and the personal care industries.” |
| *Eureka Institute* alumni | <https://eurekainstitute.org/> | “… a Network of multidisciplinary translational professionals who create a global community of Translational Medicine professionals to advance the application of biomedical innovation for the tangible benefit of patients and society as a whole.” |
| *Apollo Society* | <https://www.apollosociety.eu/> | “… an international collaborative project … with the aim of supporting students to develop the knowledge, skills, and experience that will support a career in the field of translational medicine.” |
| *SingHealth* | <https://www.singhealth.com.sg/> | “… a network of acute hospitals, national specialty centres, community hospitals and polyclinics … to drive the transformation of healthcare and provide affordable, accessible and quality healthcare.” |
| *Institute of Medical Biology A*Star* | <https://www.a-star.edu.sg/imb/> | “… focusses its research portfolio on the critical and challenging interface between basic science and medicine … to understand mechanisms underlying human disease, so that we may discover new and effective approaches to combating illness and promoting wellbeing.” |
| *Utrecht Institute for Pharmaceutical Sciences* | <https://www.uu.nl/en/research/utrecht-institute-for-pharmaceutical-sciences-uips> | “… focuses on processes around discovery, development, and use of drugs and medical nutrition using molecular and technological principles from the Natural and Life Sciences.” |

^a^ The Uniform Resource Locators (URLs) for the websites were copied on the 30^th^ of May 2019.

^b^ Descriptions are shortened texts from the websites.

## Supplementary Figures

**Supplementary figure 1.** Occupation, age and geographical location and attitude to social media.

Proportion of respondents of each occupation (**A**), age group (**B**) and geographical location (**C**) that answered NO, YES or MAYBE to the Q6 out of total respondents per occupation, age group and geographical location accordingly.


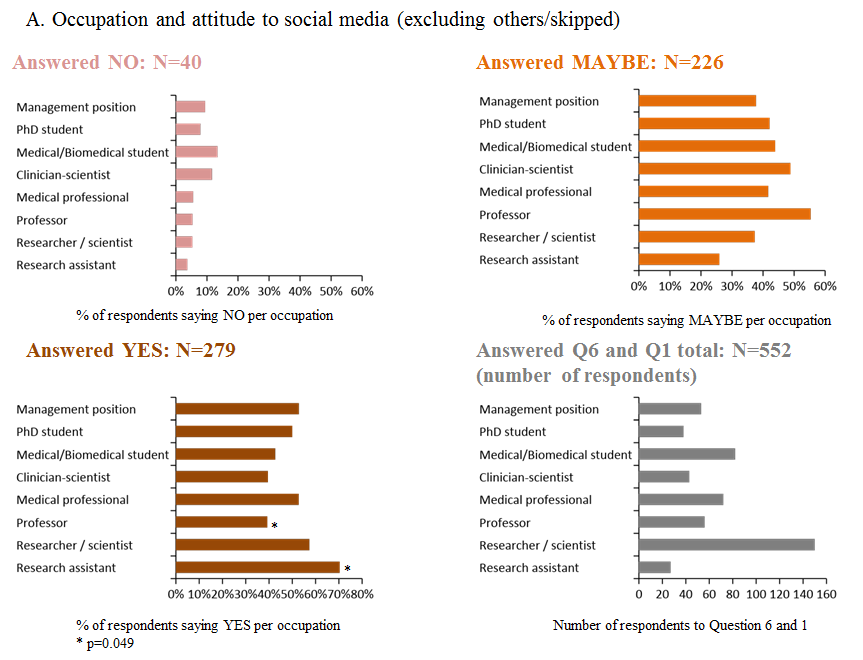


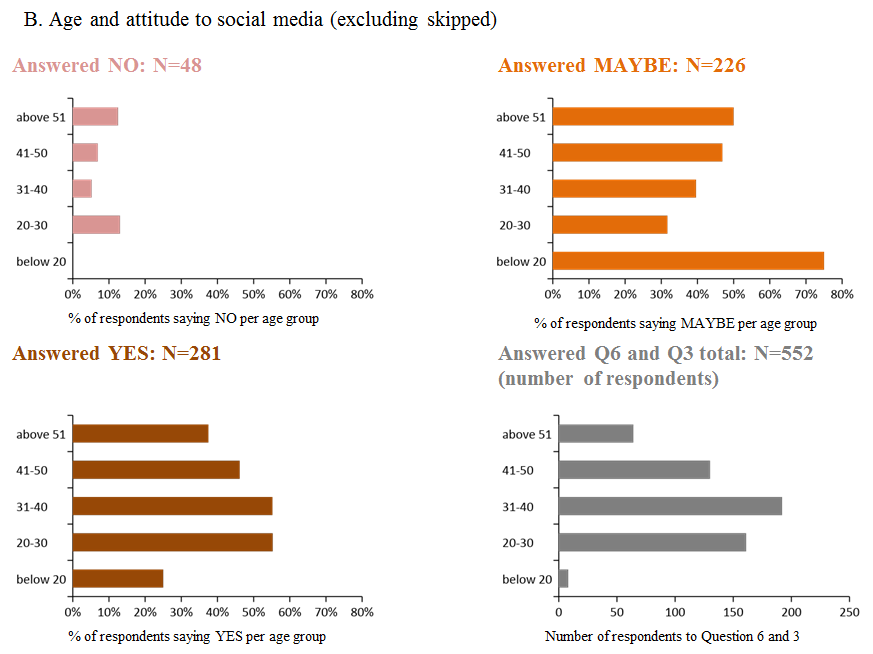


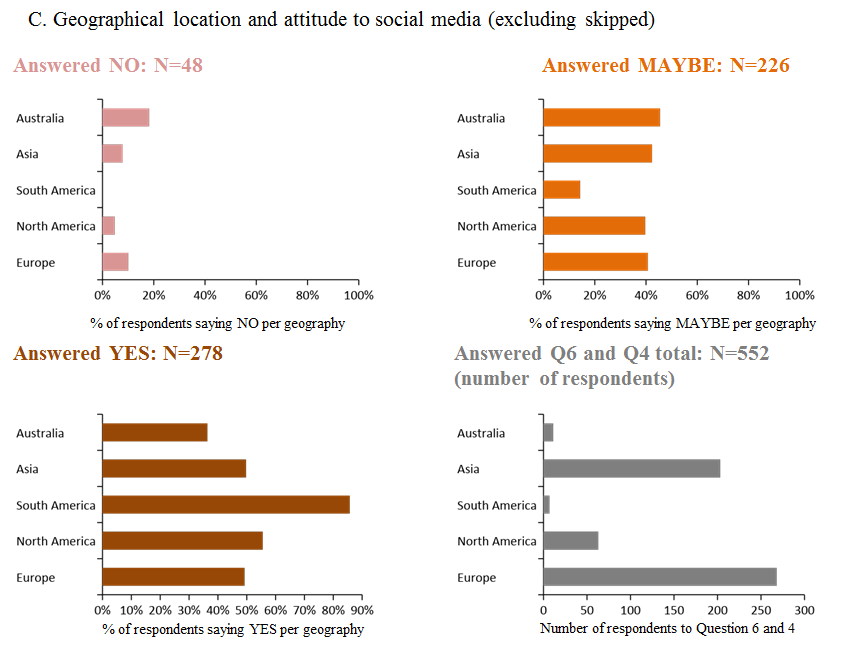


**Supplementary figure 2.** Familiarity with *Chatbot*.

Proportion of respondents of each occupation (**A**), workplace (**B**), age group (**C**) and geographical location (**D**) that answered NEVER HEARD; YES, BUT NEVER USED or YES, I HAVE USED chatbot to the Q11 out of total respondents per occupation, workplace, age group and geographical location accordingly.


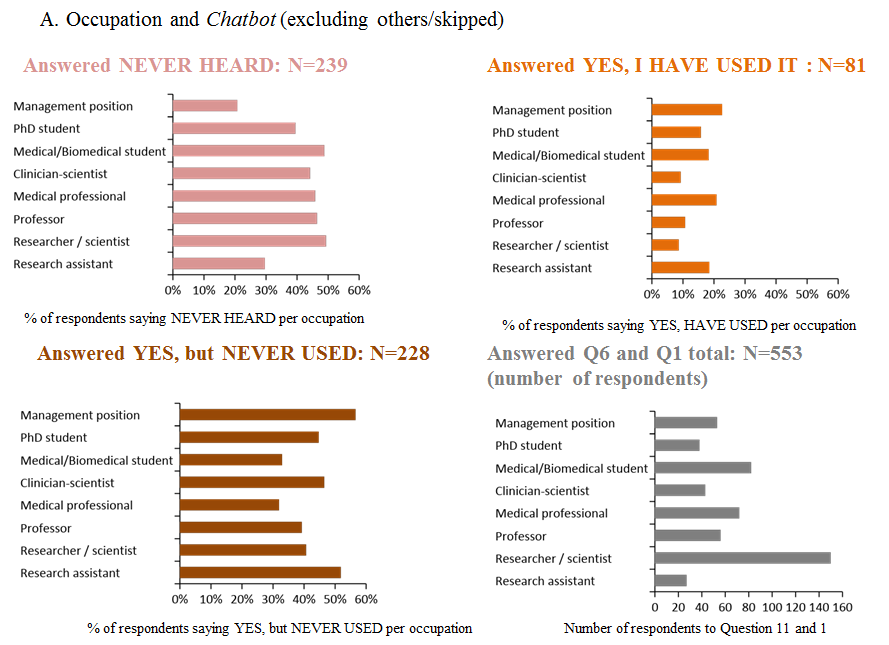


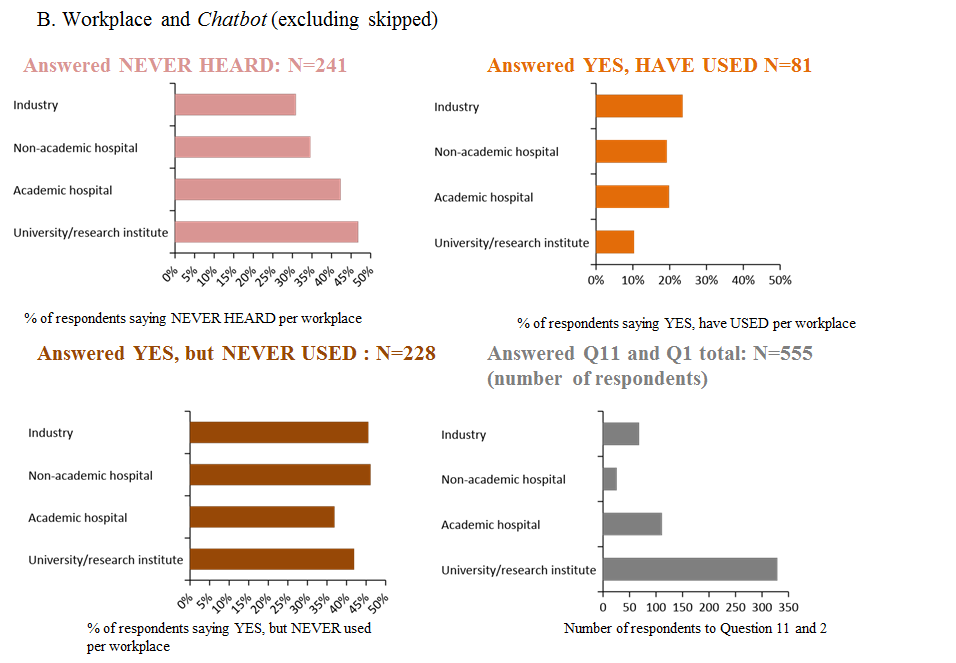


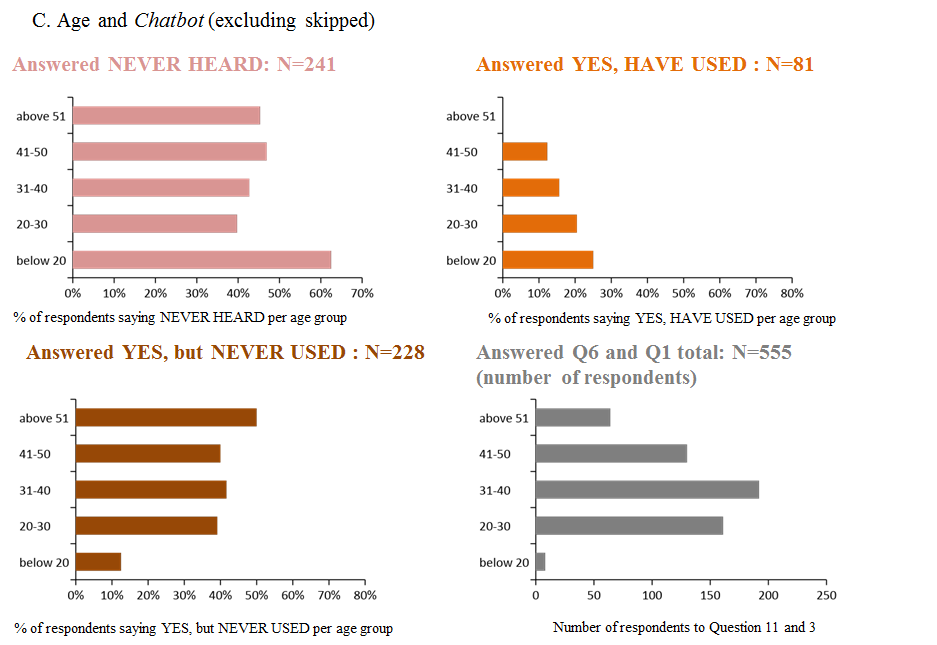


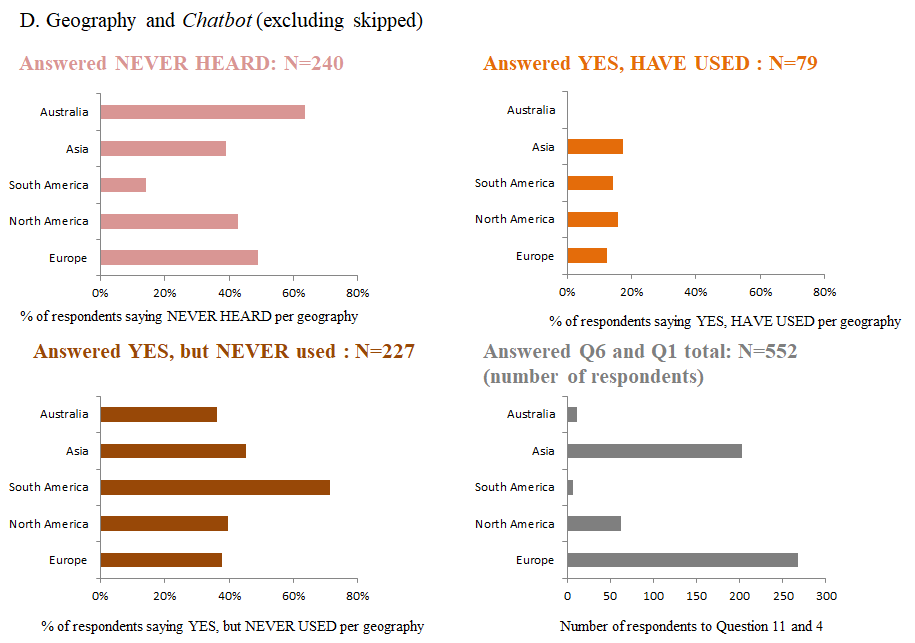

Supplement: Supplementary file 1 [file Data_Sheet_1.docx]
